# Predicting SARS-CoV-2 infection among hemodialysis patients using multimodal data

**DOI:** 10.3389/fneph.2023.1179342

**Published:** 2023-06-02

**Authors:** Juntao Duan, Hanmo Li, Xiaoran Ma, Hanjie Zhang, Rachel Lasky, Caitlin K. Monaghan, Sheetal Chaudhuri, Len A. Usvyat, Mengyang Gu, Wensheng Guo, Peter Kotanko, Yuedong Wang

**Affiliations:** ^1^ Department of Statistics and Applied Probability, University of California, Santa Barbara, CA, United States; ^2^ Renal Research Institute, New York NY, United States; ^3^ Fresenius Medical Care, Global Medical Office, Waltham, MA, United States; ^4^ Division of Nephrology, Maastricht University Medical Center, Maastricht, Netherlands; ^5^ Department of Biostatistics, Epidemiology and Informatics, University of Pennsylvania Perelman School of Medicine, Philadelphia PA, United States; ^6^ Icahn School of Medicine at Mount Sinai, New York NY, United States

**Keywords:** COVID-19, hemodialysis, machine learning, prediction, XGBoost

## Abstract

**Background:**

The coronavirus disease 2019 (COVID-19) pandemic has created more devastation among dialysis patients than among the general population. Patient-level prediction models for severe acute respiratory syndrome coronavirus 2 (SARS-CoV-2) infection are crucial for the early identification of patients to prevent and mitigate outbreaks within dialysis clinics. As the COVID-19 pandemic evolves, it is unclear whether or not previously built prediction models are still sufficiently effective.

**Methods:**

We developed a machine learning (XGBoost) model to predict during the incubation period a SARS-CoV-2 infection that is subsequently diagnosed after 3 or more days. We used data from multiple sources, including demographic, clinical, treatment, laboratory, and vaccination information from a national network of hemodialysis clinics, socioeconomic information from the Census Bureau, and county-level COVID-19 infection and mortality information from state and local health agencies. We created prediction models and evaluated their performances on a rolling basis to investigate the evolution of prediction power and risk factors.

**Result:**

From April 2020 to August 2020, our machine learning model achieved an area under the receiver operating characteristic curve (AUROC) of 0.75, an improvement of over 0.07 from a previously developed machine learning model published by Kidney360 in 2021. As the pandemic evolved, the prediction performance deteriorated and fluctuated more, with the lowest AUROC of 0.6 in December 2021 and January 2022. Over the whole study period, that is, from April 2020 to February 2022, fixing the false-positive rate at 20%, our model was able to detect 40% of the positive patients. We found that features derived from local infection information reported by the Centers for Disease Control and Prevention (CDC) were the most important predictors, and vaccination status was a useful predictor as well. Whether or not a patient lives in a nursing home was an effective predictor before vaccination, but became less predictive after vaccination.

**Conclusion:**

As found in our study, the dynamics of the prediction model are frequently changing as the pandemic evolves. County-level infection information and vaccination information are crucial for the success of early COVID-19 prediction models. Our results show that the proposed model can effectively identify SARS-CoV-2 infections during the incubation period. Prospective studies are warranted to explore the application of such prediction models in daily clinical practice.

## 1 Introduction

In December 2019, pneumonia cases of unknown cause emerged in Wuhan, China. Soon the virus was identified as a type of coronavirus named severe acute respiratory syndrome coronavirus 2 (SARS-CoV-2) (1). The resulting acute respiratory disease was named coronavirus disease 2019 (COVID-19). Owing to its highly contagious nature, SARS-CoV-2 soon spread across the globe. As of 3 January 2023, according to the WHO (2), there have been 655,689,115 confirmed cases of COVID-19 (as reported to the WHO) worldwide, including 6,671,624 deaths, equating to a death rate of over 1% among the general population.

Because of older age and multiple comorbidities, dialysis patients are at higher risk of serious complications and death from COVID-19. A greater than 10% case fatality is observed among dialysis patients in different studies (3–5). Considering that patients on maintenance hemodialysis typically have an impaired immune function and are at a higher risk from COVID-19 than the general population, special care is required. Safety procedures have been implemented in dialysis centers (e.g., temperature screenings, universal masking, isolation treatments) to control the spread of SARS-CoV-2 and avoid outbreaks. Specifically, all patients and staff with an elevated body temperature or flu-like symptoms or those who have been exposed to COVID-19 are considered “patients under investigation” (PUI). PUI undergo multiple reverse transcription-polymerase chain reaction (RT-PCR) tests for the detection of SARS-CoV-2 and are treated in dedicated isolation areas (rooms, shifts, or clinics). Although these safety procedures mitigate the rapid spread of SARS-CoV-2 within the dialysis community, they add a significant burden to daily clinic operations.

In the general population, machine learning prediction models have been applied successfully and have reduced economic burden and pandemic control costs (6–9). These COVID-19 prediction models can provide a supportive diagnosis of COVID-19 and prediction of mortality risk and severity using readily available electronic health records (8, 10–12). These efforts add another layer of protection for the general public on top of standard epidemic control procedures, such as social distancing and isolation.

Among the dialysis community, the application of such Artificial Intelligence (AI) supported solutions is still very limited. To alleviate the challenges imposed on daily clinic operations, machine learning prediction models were studied (13, 14). One advantage of adopting these AI models is the possibility of a swift response. AI models can aggregate patient information to detect SARS-CoV-2 infection several days before the RT-PCR test result is available [e.g., 3 days ahead (13)]. The combination of different data sources allows the discovery of features specific to dialysis patients other than general symptoms of COVID-19 (e.g., fever and coughing). Therefore, it may be possible to detect asymptomatic patients during the incubation period.

As the COVID-19 pandemic evolves, it is unclear whether or not previously identified predictors [e.g., residing in a nursing home in (14, 15), clinical and laboratory parameters in (15–17)] are still predictive and previously built machine learning models [e.g., XGBoost in (13)] are still effective for the early detection of COVID-19 cases. Not only has the original virus undergone mutations that have resulted in multiple variants with different clinical presentations (18–20), but also the social environment has significantly changed. For example, lockdowns and social distancing rules have been lifted and vaccination programs have been implemented. Therefore, in contrast to previous studies, we leveraged multiple data sources to study how these changes affected COVID-19 prediction modeling over a much longer period, that is, from January 2020 to February 2022. A longer study period and versatile data sources allowed us to explore the continuous dynamics of COVID-19 prediction and thus provide more reliable and time-tested insights. Ultimately, by combining these insights with AI modeling, we hope to reduce the frequency of false-positive and false-negative predictions, and, consequently, assist dialysis clinics with improving operational efficiency.

## 2 Materials and methods

### 2.1 Data collection

Fresenius Kidney Care (FKC) is a large dialysis organization that comprises about 2,400 dialysis clinics in all but one state in the United States and provides dialysis treatments for approximately one-third of all US dialysis patients. Clinical, treatment, and laboratory information are routinely collected and stored electronically. We identified FKC patients with treatment records from November 2019 to March 2022. Patients suspected of having a SARS-CoV-2 infection at the outpatient dialysis clinics universally underwent RT-PCR testing to diagnose COVID-19. Demographic and socioeconomic information, such as age, dialysis vintage, race, gender, education, employment status, and comorbidities, including hypertension, diabetes, congestive heart failure, and chronic obstructive pulmonary disease, were extracted for each patient. Vaccination information such as the vaccine type (Pfizer, Moderna, or Johnson & Johnson) and administration date were recorded for each patient. Clinical data such as pre- and post-dialysis body temperature, pre- and post-dialysis systolic blood pressure, and interdialytic weight gain (IDWG), treatment data such as treatment time, ultrafiltration volume, ultrafiltration rate, and Kt/V, and intradialytic data, such as intradialytic blood pressure, heart rate, and ultrafiltration rate, were extracted from electronic health records. Laboratory variables such as creatinine, blood urea nitrogen (BUN) and albumin levels, and neutrophil-to-lymphocyte ratio were measured about once a month; hemoglobin levels were measured weekly.

Based on each patient’s home zip code, their county-level infection information (including daily new COVID-19 cases and daily COVID-19 deaths) was extracted from the New York Times COVID-19 tracker (21). Other county information, including total population, population density, and percentage of population in poverty, was obtained from the Census Bureau (22). Another feature, “percentage of contracting (PoC) COVID-19”, was estimated for each county in the US using a COVID-19 transmission model (23), which represented the daily risk of a susceptible individual contracting COVID-19 in that county.

This study was performed under a protocol reviewed by the Western Institutional Review Board (WIRB; protocol #20212859). WIRB determined that this analysis of deidentified patient data was exempt and did not require informed consent. The analysis was conducted in accordance with the Declaration of Helsinki.

### 2.2 Confirmed cases and controls

**Figure 1:**
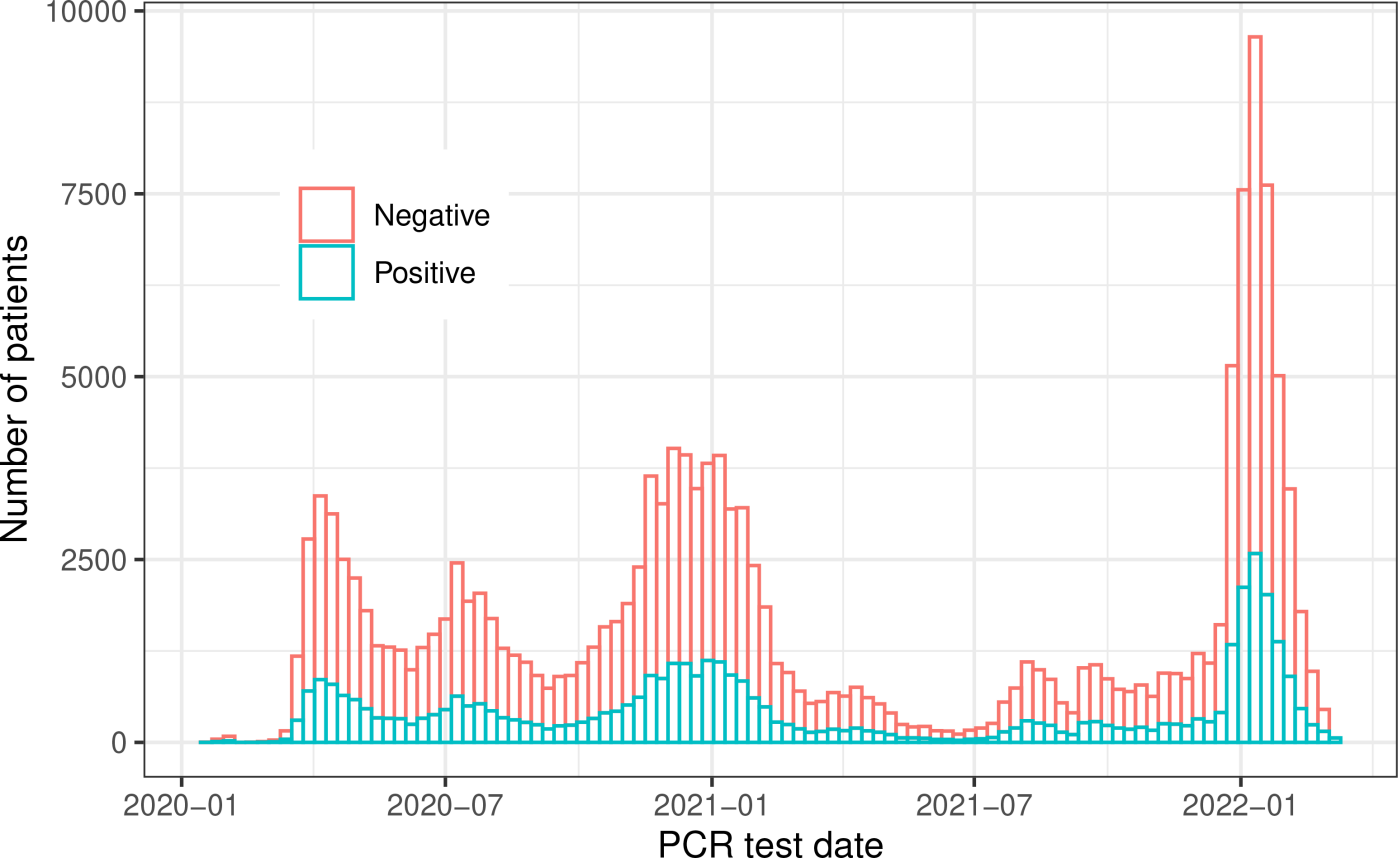
Patients’ distribution across the whole study period.

We identified 41,390 COVID-19-positive dialysis patients between 21 January 2020 and 28 February 2022. These positive patients had at least one confirmed positive RT-PCR COVID-19 test during the study period. Only the first confirmed positive date was used in this study. A total of 115,510 negative patients were randomly sampled from all active FKC patients. These COVID-19-negative patients had either a negative or no RT-PCR test during the observation period. Random sampling was performed such that the number of negative patients was approximately three times the total number of positive patients (**Figure 1**).

We defined a patient’s index date as the date of the positive RT-PCR test. For patients who had never reported a positive RT-PCR result, the index date was randomly sampled from the positive patients’ index dates.

We included only patients with (1) at least one hemoglobin test done both 1–14 days and 31–60 days before the individual’s prediction date (i.e., 3 days before the index date) and (2) at least one dialysis treatment done both 1–7 days and 31–60 days preceding the prediction date. This was done to ensure that we included only patients who were active, as hemoglobin measurements are done weekly among FKC in-center dialysis patients.

### 2.3 Data processing and feature engineering

**Figure 2:**
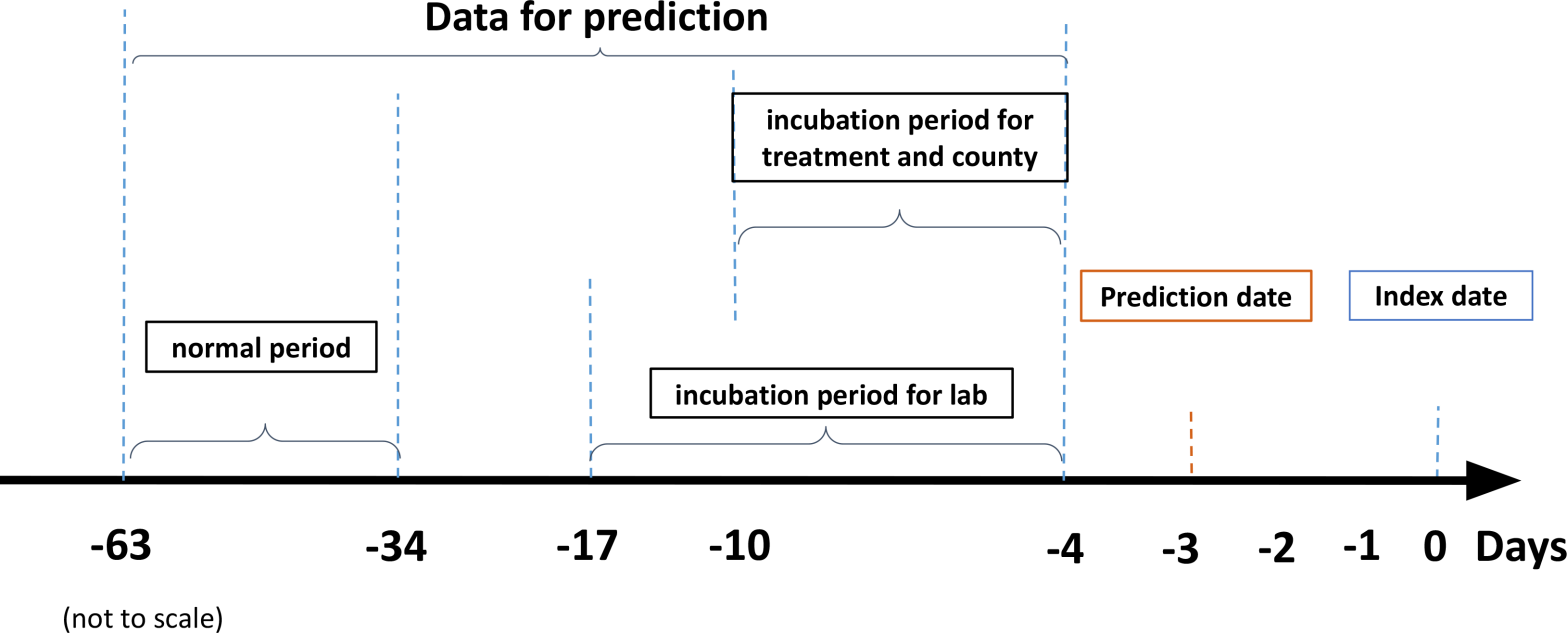
Data extraction and prediction timeline.

We followed a similar timeline setup to (13). Specifically, we used data from only up to 4 days (see **Figure 2**) before the index date (expected RT-PCR test date). First, we eliminated outliers from laboratory and treatment measures, which were likely due to manual input errors (e.g., body temperature less than 70°F or greater than 120°F). Second, we created features by taking an average of a variable over two different periods, the normal period and the incubation period. We also used the difference between the mean value in the normal period and the incubation period (the variable name is followed by “_diff”). For treatment and county infection variables, the incubation period was set to 1–7 days before the prediction date. For laboratory measurements, the incubation period was set to 1–14 days before the prediction date due to its less frequent schedule. The normal period is 31–60 days before the prediction date for every variable. Third, since vaccinal immunization decays over time (24), we also calculated the time from the prediction date to the latest vaccination date to reflect this effect. Lastly, the infection or death rate (number of infections or deaths per million people due to COVID-19) at the county level was also calculated to reflect the local epidemic characteristics.

### 2.4 Models

#### 2.4.1 Training and testing

**Figure 3:**
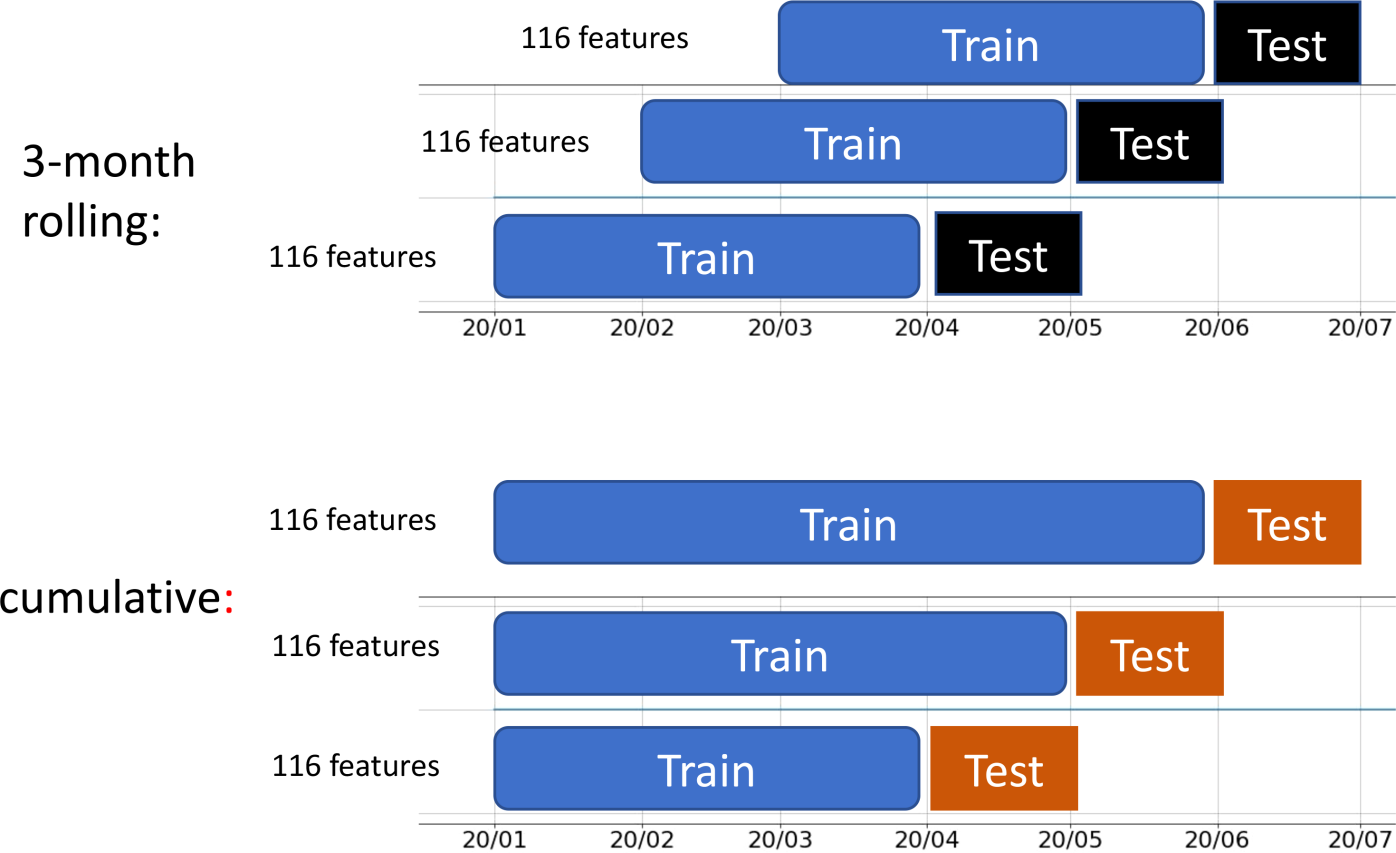
Two types of training strategy: 3-month rolling and cumulative.

We selected 116 features, including demographic, treatment, laboratory, and local county variables, up to 4 days before the index date. We used these features to predict the risk of a SARS-CoV-2 infection being identified in the next 3 or more days (i.e., on or after the index date). We used a monthly updating strategy to emulate implementation in dialysis clinics. For example, for the prediction in August 2020, we used data before 1 August 2020 as the training sample. Thus the prediction performance on August 2020 was out of the training sample and close to the real-world performance. We compared two types of training as shown in **Figure 3**, one that used only data within 3 months before the testing period, and another that used all data from the beginning of 2020. The hyperparameters of XGBoost were tuned on the training dataset using cross-validation. The training used binary cross-entropy as the loss function, with weights set as the ratio of positive to negative patients to solve the class imbalance problem.

#### 2.4.2 Evaluation metric

We used the monthly out-of-sample area under the receiver operating characteristic curve (AUROC) to evaluate the performance over the period April 2020 to February 2022. We also calculated the overall AUROC and precision–recall curve (PRC) with aggregated monthly predictions.

#### 2.4.3 Feature importance

We used SHapley Additive exPlanations (SHAP) values to identify the influential variables on monthly testing predictions (25, 26). For each specific prediction, the SHAP value was computed for every variable, which measures how much the predicted value is affected by each variable used in the XGBoost model. The overall feature importance of each variable can be quantified by the mean absolute value of SHAP values for each variable across all observations.

## 3 Results

### 3.1 Model performance

**Figure 4:**
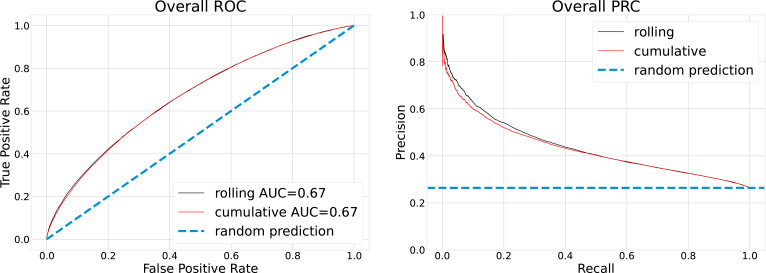
Overall testing performance is calculated with aggregated monthly predictions.

**Figure 5:**
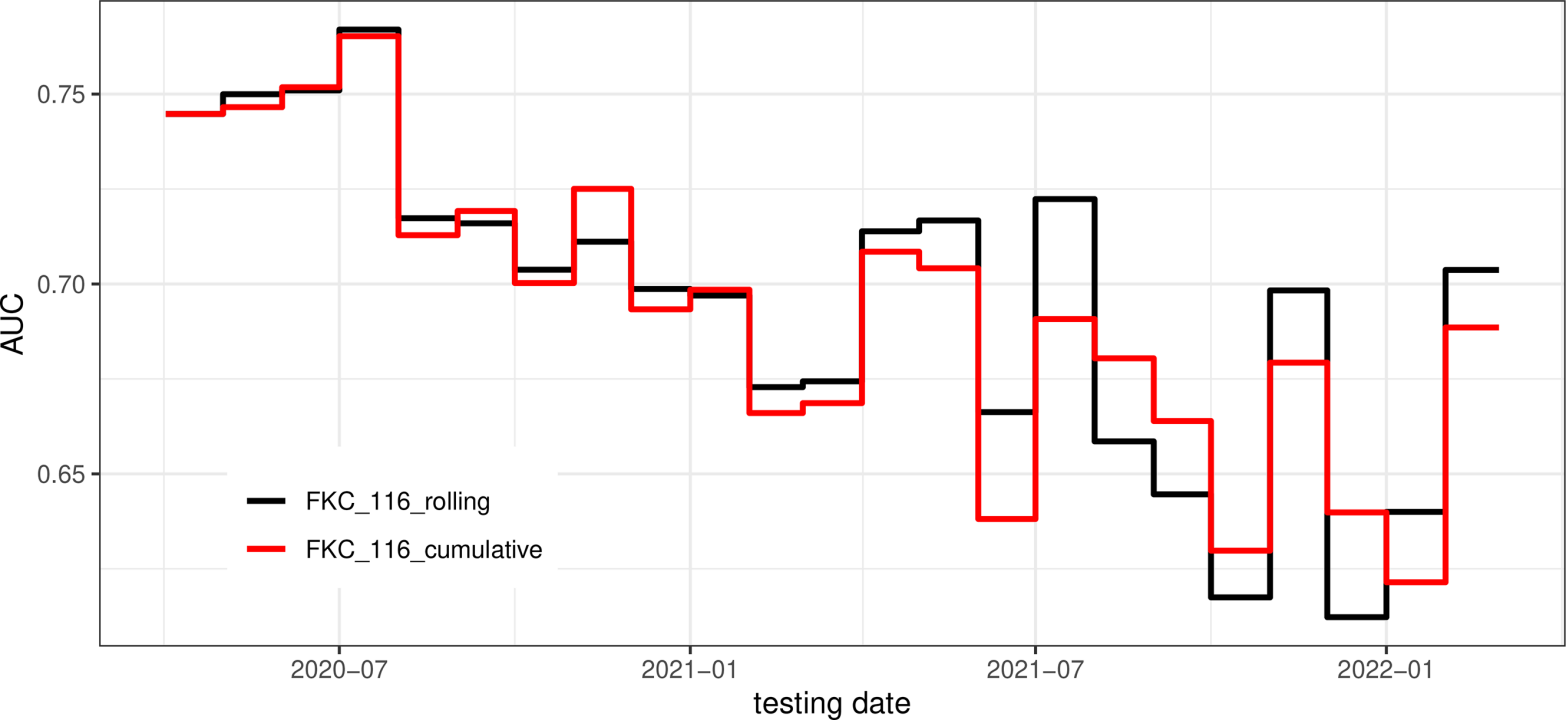
Monthly AUROC.

To assess the impact of different training strategies on model performance, we compared overall AUROC and PRC in **Figure 4**, and monthly AUROC in **Figure 5**.

Overall, testing performance in **Figure 4** was calculated by aggregating all monthly predictions. For instance, overall precision was computed as the ratio of correctly predicted positive COVID-19 cases to the total number of positive COVID-19 cases over the entire testing period, that is, from April 2020 to February 2022. As shown in **Figure 4**, there is minimal difference between the 3-month rolling and cumulative models. In both cases, with the false-positive rate fixed at 20%, the true-positive rate is slightly above 40%.

In **Figure 5**, we find the two training strategies exhibit only slight variations in their monthly performance. The 3-month rolling models are more responsive to recent changes, such as sudden waves. In **Figure 5**, the AUROC started at around 0.75 and dropped slightly to 0.70 on August 2020. After that, the performance fluctuates between 0.60 and 0.70. Multiple reasons may cause this trend of performance degradation. First, predictive features may become unproductive over time for various reasons, which will be discussed later when investigating feature importance. Second, as the reopening policy was rolled out, pandemic characteristics have changed. Third, data quality is degrading over time due to under-reporting because of asymptomatic cases and less frequent updates of county infection reports by CDC^^1^^.

In the study of Monaghan et al. (13), FKC patients’ data from 27 February 2020 to 8 September 2020 was used to build a machine learning model for early prediction of COVID-19 cases. Their model focused on biological changes in clinical biomarkers and achieved a testing AUROC of 0.68. Compared with Monaghan et al. (13), we achieved a higher testing AUROC at around 0.75 (**Figure 5**) before 31 August 2020. The performance improvement is due to additional data being included; local county infection information in particular played an important role. After 2021, as vaccination was rolled out, we identified that vaccination become a crucial predictor, which only our study was able to investigate..

### 3.2 Feature importance

**Figure 6:**
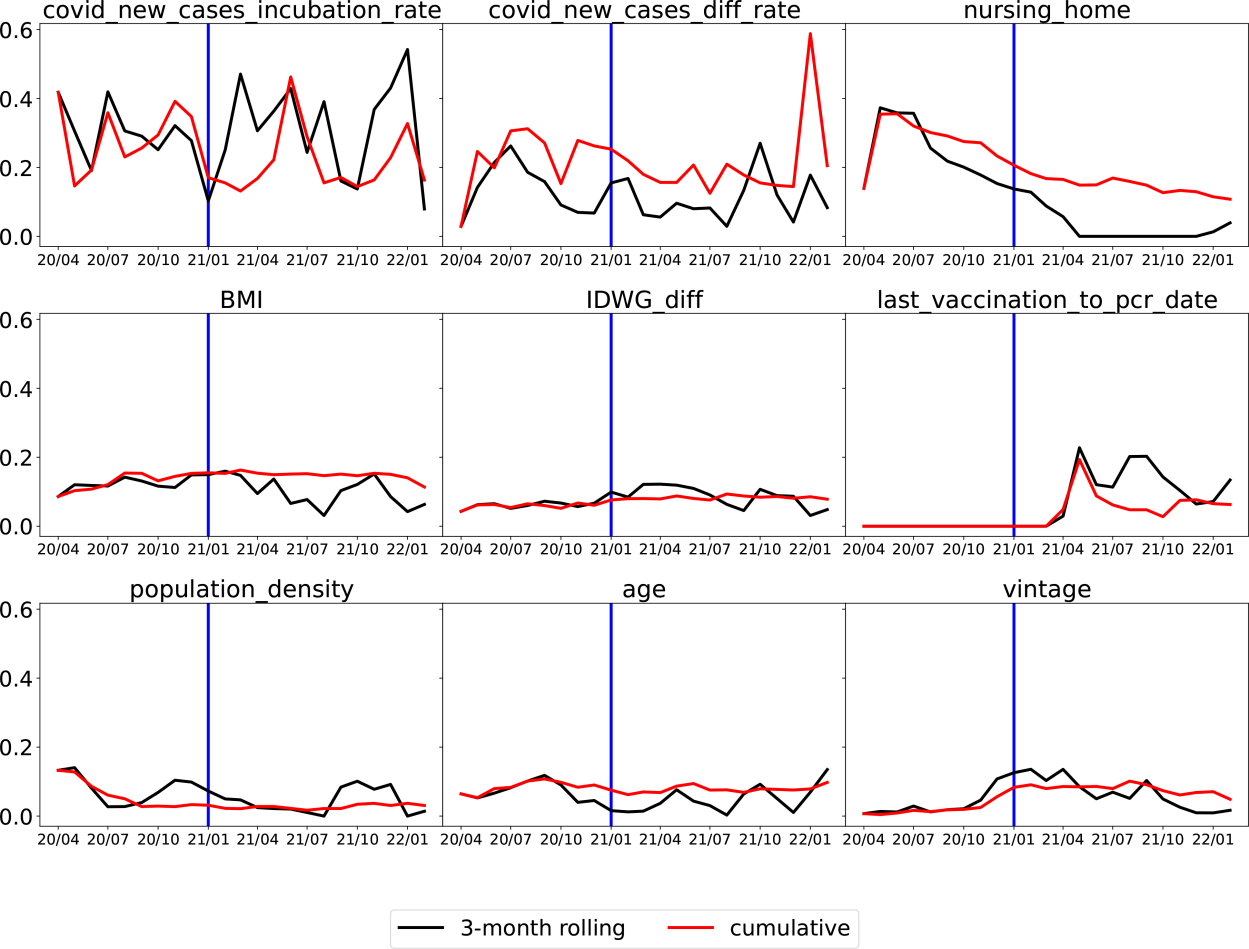
Monthly average absolute SHAP value for the top nine important features. For each feature, the black line is for the 3-month rolling model, and the red line is for the cumulative model. The vertical blue line at the end of 2020 is a separation of whether or not vaccination is available (U.S. HHS, Vaccination in the US began on 14 December 2020). (Note that the x-axis is the date in “yy/mm” format).

After ranking the mean absolute value of SHAP values, the top 40 features were identified and summarized. They are shown in **Tables 1**, **2**. The top nine features are shown in **Figure 6**.

**Table 1:** Demographics and categorical features of hemodialysis patients with and without a severe acute respiratory syndrome coronavirus 2 (SARS-CoV-2) infection.

Variable	Unaffected patients	COVID-19-positive patients
Number of patients on HD	115,510	41,503
Male, n (%)	67,717 (59)	22,857 (55)
Hispanic or Latino, n (%)	15,748 (14)	7,625 (19)
Diabetes, n (%)	48,928 (45)	19,256 (49)
Nursing home, n (%)	6,025 (6)	5,290 (15)
Race, *n* (%)		
American Indian or Alaska Native	877 (1)	587 (1)
Asian	4,063 (4)	988 (2)
Black people or African American	38,664 (35)	13,632 (34)
Native Hawaiian or Other Pacific Islander	1,285 (1)	481 (1)
White people	65,717 (59)	24,434 (61)
Education, n (%)		
8 or less years of school	8,592 (7)	4,289 (10)
Current student	78 (0)	31 (0)
GED	2,951 (3)	1,142 (3)
Graduated from 2- or 4-year college	17,563 (15)	4,871 (12)
Graduated high school	44,257 (38)	16,113 (39)
Graduate school	4,960 (4)	1,135 (3)
More than 8 years but less than 12 years	14,252 (12)	6,390 (15)
Some college	18,677 (16)	6,156 (15)
Vocational/technical school	4,074 (4)	1,364 (3)
Other	106 (0)	12 (0)

COVID-19, coronavirus disease 2019; GED, general educational development.

**Table 2:** Numerical features in top 40 most important features of hemodialysis patients with and without a severe acute respiratory syndrome coronavirus 2 (SARS-CoV-2) infection.

Variable	Unaffected patients,	COVID-19-positive patients,
	mean ± SD	mean ± SD
Age (years)	64.1 ± 4.15	62.84 ± 4.32
Height (cm)	168.96 ± 1.37	168.21 ± 1.38
BMI (kg/m^2^)	28.82 ± 7.49	29.77 ± 7.86
Number of days since last vaccination	123.46 ± 99.16	144.13 ± 104.49
Dialysis vintage (days)	1,481.85 ± 501.48	1,530.17 ± 466.68
County-level local information per million population		
Daily infected COVID cases	642.25 ± 863.73	755.07 ± 868.42
Change in daily infected COVID cases	380.69 ± 872.24	487.99 ± 883.68
Change in daily COVID death	186.15 ± 98.99	197.52 ± 76.73
Treatment information		
Pre-HD body temperature (°F)	97.36 ± 0.61	97.45 ± 0.64
Post-HD body temperature (°F)	97.45 ± 0.52	97.51 ± 0.56
Change in post-HD body temperature (°F)	–0.01 ± 0.45	0.05 ± 0.49
Change in pre-HD weight loss (kg)	–0.21 ± 2.42	–0.4 ± 2.51
Change in weight (kg)	0.04 ± 13.54	0.04 ± 15.42
Change in IDWG (kg)	0.02 ± 0.87	–0.11 ± 0.96
Change in pre-HD pulse (BPM)	0.04 ± 7.46	0.76 ± 7.51
Change in Post-HD pulse (BPM)	–0.05 ± 7.34	0.98 ± 7.60
Change in max-HD pulse (BPM)	0.07 ± 8.05	1.07 ± 8.34
Min-HD pulse (BPM)	65.8 ± 10.52	66.8 ± 10.60
Max-HD sitting SBP (mmHg)	155.58 ± 22.68	158.13 ± 22.89
Post-HD sitting SBP (mmHg)	139.33 ± 21.03	141.4 ± 21.38
Change in pre-HD sitting SBP (mmHg)	0.19 ± 15.69	–0.87 ± 16.56
Change in pre-HD sitting DBP (mmHg)	0.1 ± 9.07	–0.43 ± 9.43
Laboratory measurements		
Albumin (g/dL)	3.82 ± 0.42	3.73 ± 0.44
Calcium (mg/dL)	8.94 ± 0.68	8.85 ± 0.69
Change in % of monocytes	0 ± 1.24	0.15 ± 1.42
Change in WBC count (10^10^/L)	–0.05 ± 2.92	–0.14 ± 2.05
Hgb (g/dL)	10.73 ± 1.25	10.64 ± 1.23
TSAT (%)	32.63 ± 14	31.7 ± 14.18
URR	74.58 ± 6.63	74.82 ± 6.34
WBC count (10^10^/L)	6.96 ± 3.59	6.85 ± 3.00
% of eosinophils	4.33 ± 2.66	4.09 ± 2.62

COVID-19, coronavirus disease 2019; BMI, body mass index; HD, hemodialysis; IDWG, interdialytic weight gain; TSAT, transferrin saturation; URR, urea reduction ratio.

**Figure 7:**
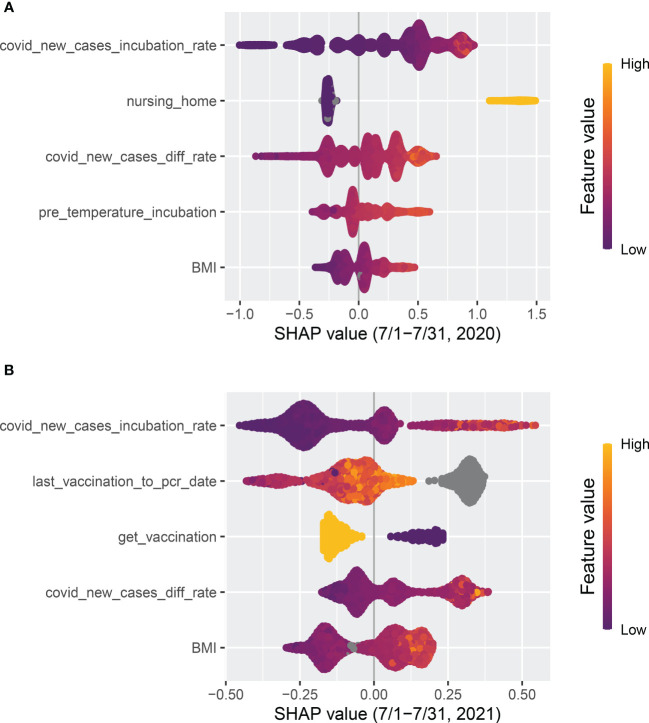
Top five features ranked by absolute SHAP value. **(A)** The model was trained with the data from 1 April to 30 June in 2020 and tested on data from 1 July to 31 July, 2020. **(B)** The model was trained with data from 1 September to 30 November in 2021 and tested on data from 1 December to 31 December, 2021.

The top two features that remained important throughout the whole time period were the average number of new COVID-19 cases per million population in the incubation period (“covid_new_cases_incubation_rate”) and the difference in the average number of new COVID-19 cases per million population between the incubation period and the normal period (covid_new_cases_diff_rate). In **Figure 7**, “covid_new_cases_incubation_rate” and “covid_new_-cases_diff_rate” have a positive correlation with COVID-19 cases. These two features were derived from local county COVID-19 cases reports. Another important feature of local information is population density (“population_density”), as high population densities can increase the risk of spreading SARS-CoV-2. In areas with high population densities, such as cities or densely populated neighborhoods, it can be difficult to maintain physical distancing and limit close contact with others.

Whether or not a patient lives in a nursing home (“nursing_home”) was a robust top predictor before 2021, but its significance has gradually declined. Following the implementation of vaccination in 2021, a substantial decrease was observed. As shown in **Figure 7**, “nursing_home” was ranked as the second most significant factor that positively correlated with COVID-19 cases in July 2020. However, by July 2021 it had dropped out of the top five features.

Clinical (treatment) and demographic information such as body mass index (“BMI”), interdialytic weight gain between the incubation period and normal period (“IDWG_diff”), and vintage appeared among the top nine features, which confirms the findings in Chaudhuri et al. (16) that clinical and laboratory variables are predictive. As shown in **Figure 7**, pre-dialysis body temperature during the incubation period (“pre_temperature_incubation”) was also identified as one of the top five predictive features in July 2020 and had a positive correlation with COVID-19 cases. However, similar to “nursing_home”, it had dropped out of the top five features by July 2021.

The variable “last_vaccination_to_pcr_date” is defined as the difference between a patient’s prediction date and the latest vaccination date. As illustrated in **Figure 7**, “last_vaccination_to_pcr_date” emerged as a significant predictor in the middle of 2021, ranking first in importance, and had a positive correlation with COVID-19 cases. Specifically, a missing or large value in “last_vaccination_to_pcr_date” implied that the patient had a higher chance of being identified as a positive COVID-19 case. However, by the end of 2021 its significance was reduced **Figure 6**, possibly due to a decrease in the efficacy of vaccination-induced antibodies over time. Similarly, another vaccination-related feature, “get_vaccination”, which was defined as whether or not a patient had received vaccines before the prediction date, has a negative correlation with positive COVID-19 cases, as shown in **Figure 7** compare before after vaccination.

Comparing the two training strategies, the 3-month rolling models (represented by black lines) generally produced rougher curves for SHAP values, as they were quicker to respond to rapid changes. In contrast, cumulative models (represented by red lines) utilized accumulated data, making them less responsive to changes such as the introduction of vaccination.

## 4 Discussion

We have successfully developed a machine learning model that utilizes multiple data sources to detect early COVID-19 infections in maintenance hemodialysis patients. We demonstrated that the proposed machine learning model achieved clinically meaningful performance by monthly testing throughout the COVID-19 pandemic. Overall, the model was able to identify 40% of COVID-19 patients (with a 20% false-positive rate) before they were identified by an RT-PCR COVID-19 test. This can significantly aid dialysis clinics in preventing the spread of the virus by implementing targeted procedures for identified patients.

More importantly, apart from the patient’s laboratory and treatment information (e.g., body temperature) used by Monaghan et al. (13), we identified two other sources of information that are more critical for such prediction models. The first is local county infection data provided by CDC. County-level COVID-19 infection information reflects how likely SARS-CoV-2 is to spread within the patient’s community. The second is the patient’s vaccination record, which reflects how likely SARS-CoV-2 will infect a patient after contact. As the dynamics of COVID-19 change, previously important features, such as whether or not a patient lives in a nursing home, become less predictive.

There are limitations to our study. First, the positive COVID-19 diagnosis labels are limited to the positive patients’ specific RT-PCR test date. As the COVID-19 pandemic is a continuous and dynamic process, there are dates before and after the RT-PCR test date that should also be annotated as positive labels. However, without enough information, it is difficult to select these periods. Furthermore, the timing of the RT-PCR test relative to infection can vary based on symptom presentation and other factors, adding additional variability to the RT-PCR test dates. It is worth further investigating the cutoff dates to further improve COVID-19 early detection. Second, as limited by anticipated data availability and model integration into clinical systems, the prediction date was set to 3 days before the index date. Ideally, with real-time data aggregation, one could set the prediction date to the index date. This will likely improve the detection performance as the most recent laboratory and treatment data can be used. In addition, more advanced methods such as deep learning could potentially further improve the accuracy of COVID-19 detection (27–30). This avenue will be explored in future research endeavors.

## Data availability statement

The original contributions presented in the study are included in the article/supplementary material. Further inquiries can be directed to the corresponding author.

## Ethics statement

The studies involving human participants were reviewed and approved by the Western Institutional Review Board. Written informed consent for participation was not required for this study in accordance with the national legislation and the institutional requirements.

## Author contributions

YW, PK, HZ, WG, MG, and LU conceived and designed the study. RL and SC collected the data. JD, HL, and XM performed the statistical analysis and wrote the manuscript’s original draft. CM provided advice on the prediction model published by Kidney360 in 2021. All authors contributed to the article and approved the submitted version.

## Footnotes

1
CDC COVID-19 surveillance switched to the weekly report on October 20, 2022
